# Evaluation of time-dependent toxicity and combined effects for a series of mono-halogenated acetonitrile-containing binary mixtures

**DOI:** 10.1016/j.toxrep.2016.07.003

**Published:** 2016-07-25

**Authors:** Douglas A. Dawson, Daphne Guinn, Gerald Pöch

**Affiliations:** a Department of Biology/Toxicology, Ashland University, Ashland, OH, USA; b Department of Pharmacology and Toxicology, University of Graz, Graz, Austria

**Keywords:** Iodoacetonitrile (PubChem CID: 69356), Bromoacetonitrile (PubChem CID: 11534), Chloroacetonitrile (PubChem CID: 7856), Dibromoacetonitrile (PubChem CID: 18617), Ethyl acrylate (PubChem CID: 8821), Ethyl bromoacetate (PubChem CID: 7748), Ethyl propiolate (PubChem CID: 12182), Linalool (PubChem CID: 6549), Methyl vinyl ketone (PubChem CID: 6570), Trichloroacetonitrile (PubChem CID: 11011), Microtox^®^, Acute toxicity, Dose-addition, Independence

## Abstract

Mixture and time-dependent toxicity (TDT) was assessed for a series of mono-halogenated acetonitrile-containing combinations. Inhibition of bioluminescence in *Aliivibrio fischeri* was measured after 15, 30 and 45-min of exposure. Concentration-response (x/y) curves were determined for each chemical alone at each timepoint, and used to develop predicted x/y curves for the dose-addition and independence models of combined effect. The x/y data for each binary mixture was then evaluated against the predicted mixture curves. Two metrics of mixture toxicity were calculated per combined effect model: (1) an EC_50_-based dose-addition (AQ) or independence (IQ) quotient and (2) the mixture/dose-addition (MX/DA) and mixture/independence (MX/I) metrics. For each single chemical and mixture tested, TDT was also calculated. After 45-min of exposure, 25 of 67 mixtures produced curves that were consistent with dose-addition using the MX/DA metric, with the other 42 being less toxic than predicted by MX/DA. Some mixtures had toxicity that was consistent with both dose-addition and independence. In general, those that were less toxic than predicted for dose-addition were also less toxic than predicted for independence. Of the 25 combinations that were consistent with dose-addition, 22 (88%) mixtures contained chemicals for which the individual TDT values were both >80%. In contrast, of the 42 non-dose-additive combinations, only 2 (4.8%) of the mixtures had both chemicals with individual TDT values >80%. The results support previous findings that TDT determinations can be useful for predicting chemical mixture toxicity.

## 1 Introduction

Determining and predicting the effects of toxic substances in combination has been a focal point of recent mixture toxicity research. Via exposure profiling of reactive mixtures [1], docking-based receptor library [2] or toxicogenomics [3] studies, or examining aquatic toxicity [4], mammalian reproductive effects [5] or endocrine disruptors [6] such research efforts can improve the ability to predict mixture toxicity. Assessments of toxicity in binary [7], ternary [8] or complex chemical mixtures [9] have been common, with such toxicity having been evaluated for metals [10], pesticides [11], polycyclic aromatic hydrocarbons [12], [13], and micropollutants [14]. Conceptual studies and efforts to develop and evaluate mixture toxicity models [15], [16], [17], [18], [19], [20] have also provided approaches for improving toxicity assessment of mixtures.

Microtox^®^ is one assay often used to examine chemical mixture toxicity. This system makes use of bacteria that, through the process of quorum-sensing, produce light that can be reliably quantified by a light meter. When the bacteria are exposed to single chemicals or chemical mixtures at concentrations that exert toxicity, metabolism is adversely affected, thereby reducing the amount of light emitted. Light readings can be made prior to chemical exposure and for up to three selected timepoints after introduction of the toxicant. For the acute toxicity assay exposure can last for up to 100 min; after that point bacterial metabolism begins to wane as no nutrients are included in the reagent. Since readings can be made after chemical exposure at three selected timepoints, it is possible to assess the time-dependent toxicity of a given chemical, mixture, or environmental sample. Agents that act as non-polar narcotics tend to show inhibition of bioluminescence early on during exposure but then recover, at least partially, so that bioluminescence stabilizes or increases slightly after the initial diminution. For other chemicals toxicity continues to progress over exposure time such that bioluminescence decreases throughout exposure. These features allow the assay to be used effectively for assessing changes in toxicity over exposure time (i.e., time-dependent toxicity).

Recent studies from this lab have evaluated time-dependent toxicity (TDT) for potential value in mixture toxicity prediction using Microtox^®^. Initial studies examined mixture toxicity for binary combinations of soft electrophiles [21]; subsequently examining such toxicity in the context of chemical reactivity [22], [23]. Through binary mixture assessments of S_N_2-reactive α-halogenated acetonitriles [24], ethyl α-halogenated ethyl acetates [25], and combinations of these two groups [26] it was determined that including TDT assessments added value to mixture toxicity studies. An asymmetry parameter was incorporated into curve-fitting of single-chemical and mixture concentration-response (x/y) data to more precisely evaluate mixture toxicity against the dose-addition and independence models of combined effect [27]. Most recently TDT assessments were examined for use in predicting mixture toxicity [28].

In the latter study, it was demonstrated that taking the average TDT values of the individual chemicals in a mixture could be used to predict the TDT of the mixture [28]. This was true even when chemicals with high TDT (i.e., ≥90%) were tested with ones having low (i.e., <30%) or negative TDT despite the observed mixture TDT value being more likely to deviate from the predicted TDT value. Having previously incorporated the asymmetry parameter into curve-fitting of x/y data it became of interest to specifically assess aspects of the curve-fitting parameters for insights into the relationship between TDT and combined effect.

As noted above, α-halogenated acetonitriles are S_N_2-reactive soft electrophiles. In previous studies from this lab three mono-halogenated acetonitriles (i.e., iodoacetonitrile – IAN, bromoacetonitrile – BRAN and chloroacetonitrile – CLAN) were tested in sham combinations and with each other [24], with ethyl α-halogenated ethyl acetates [26], and with a few other organic chemicals [28]. The latter study only reported TDT values for the single chemicals and mixtures, not the specific combined effects observed. In this study, the three mono-halogenated acetonitriles (XANs) were tested in binary combination with a number of additional organic chemicals. The latter were selected to span the range of TDT values (<0%–>100%) and were compiled along with the previous XANs mixture data to fully evaluate combined effects as they relate to TDT. In order to provide a consistent basis for this assessment, the maximum effect constraint in curve-fitting was fixed at 100%. A collective summary of these results, including data for 28 combinations previously unpublished in any form, is provided herein.

## 2 Materials and methods

### 2.1 Chemicals and reagents

Chemicals used in testing (Table 1) were obtained from Sigma-Aldrich (Milwaukee, WI) at ≥95% purity and used as received. Microtox^®^ bacterial reagent, reconstitution solution and diluent were obtained from Modern Water, Inc. (New Castle, DE).

**Table 1:** Chemicals selected for testing.

Abbr.	Chemical name	CAS #^a^
3M2B	3-methyl-2-butanone	563-80-4
4NBB	4-nitrobenzyl bromide	100-11-8
BGE	butyl glycidyl ether	2426-08-6
BRAN	bromoacetonitrile	590-17-0
CLAN	chloroacetonitrile	107-14-2
DBRAN	dibromoacetonitrile	3252-43-5
DCLAN	dichloroacetonitrile	3018-12-0
DEM	diethyl maleate	141-05-9
EA	ethyl acrylate	140-88-5
EAC	ethyl acetate	141-78-6
EBAC	ethyl bromoacetate	105-36-2
ECAC	ethyl chloroacetate	105-39-5
EFAC	ethyl fluoroacetate	459-72-3
EIAC	ethyl iodoacetate	623-48-3
EP	ethyl propiolate	623-47-2
IAN	iodoacetonitrile	624-75-9
LIN	linalool	78-70-6
M2BP	methyl-2-bromopropionate	5445-17-0
MC	methyl crotonate	623-43-8
MVK	methyl vinyl ketone	78-94-4
NER	nerol	106−25-2
PN	propionitrile	107−12-0
TCLAN	trichloroacetonitrile	545−06-2

a Chemical Abstract Service registry number.

### 2.2 Toxicity testing

A routinely calibrated Microtox^®^ 500 analyzer was used to determine inhibition of bioluminescence in the marine bacterium *Aliivibrio fischeri* (formerly *Vibrio fischeri*) [29] following established procedures [26]. An experiment in this testing protocol is defined as consisting of three toxicity tests: chemical A-alone (A), chemical B-alone (B) and a “true” mixture (A + B). Some experiments were of the “sham” variety, in which two preparations of a chemical were tested alone (i.e., A_1_ and A_2_) and combined as a “mixture” (i.e., A_1_ + A_2_).

Concentration selection for each chemical was made based on results of preliminary tests and, as much as possible, designed to obtain an approximately equitoxic potency ratio (i.e., 1:1) after 30-min of exposure. At least seven concentrations were tested in duplicate (i.e., two vials per concentration) for each chemical or mixture along with a duplicated control. Nominal concentrations, corrected for density, were prepared via serial dilution. For any given experiment a single dilution factor (1.6, 1.75, 1.867, or 2.0) was used, having been selected to most effectively calculate EC_25_, EC_50_ and EC_75_ values, based on preliminary testing. The EC_50_ refers to the half-maximal effective concentration, while the EC_25_ and EC_75_ represent the one-quarter and three-quarters-maximal effective concentrations, respectively.

For each experiment, chemical A, chemical B and the mixture of A and B were tested on the same day, typically within a 4.5 h time period. Separate stock solutions of chemical A and chemical B were prepared immediately prior to testing. The mixture stock solution was prepared from the single chemical stock solutions. In testing, initial light readings were taken before chemical exposure. During exposure light readings were taken 15, 30 and 45-min after toxicant introduction. Microtox^®^ Omni software calculated the percent effect value for each vial at each exposure duration.

### 2.3 Curve fitting

Nine concentration-response (x/y) curves (i.e., three curves for chemical A: one each at 15, 30 and 45-min, along with three curves each for chemical B and the mixture at those same timepoints) were obtained from each experiment. After input to SigmaPlot^®^ (v. 11.0; Systat Software, Chicago, IL) x/y data were fitted to sigmoid curves using the 5-parameter logistic minus 1-parameter (5PL-1P) function [27]. This approach utilized four parameters: EC_50_, slope, maximum effect and asymmetry, as the minimum effect parameter had been removed from the original 5PL function within the software.

Curve fitting was performed using:

$$y=max÷{\left[1+{\left(xb÷x\right)}^{slope}\right]}^{s}$$

in which y = % effect, max = maximum effect, x = concentration, s = asymmetry. The variable xb was determined using:

$$xb=EC_{50}\times 10^{\left[\left(1÷slope\right)\times \text{log}\left(2^{\left(1÷s\right)}-1\right)\right]}$$

Initial parameters for the regressions were automatically estimated while employing three constraints: (a) EC_50_ > 0; (b) 0.1 < s < 10; and (c) max = 100. As noted above (see Introduction) maximum effect values were constrained to 100%, to provide consistency in calculating TDT values across the individual chemicals and to provide a common basis for evaluating the relationship between TDT and combined effect. For all single-chemical x/y data, EC_25_, EC_50_, EC_75_, slope, and asymmetry values were calculated at the three exposure durations.

The quality of data fitting was measured in two ways. Individual x/y curve fitting using the 5PL-1 P function was assessed by determining coefficient of determination (r^2^) values for each curve. To evaluate test-to-test consistency of the EC_50_ values for each chemical tested alone, the mean and standard deviation (S.D.) of the EC_50_ were determined and the coefficient of variation (CV) values were calculated using:

$$CV=\frac{100\times S.D.}{mean}$$

For mixture x/y data, concentrations of chemical B were converted to concentration equivalents of chemical A. The conversion factor for calculating the concentration equivalents of chemical B was determined by dividing the concentration of chemical A by the concentration of chemical B [24]. This permitted the total chemical concentration of the mixture to be made relative to those of chemical A alone, while allowing the plot of the mixture curve at any exposure duration to be graphed as mg/L concentrations for chemical A and chemical B individually. The same curve-fitting methods used for the individual chemicals were used for the mixture tests.

### 2.4 Calculation of TDT values

TDT values were calculated to quantify changes in toxicity of the individual chemicals and the mixtures over exposure time. As detailed previously [28] TDT values at various timepoints and at the 25%, 50% and 75% effect levels could be generated based on the approach of Haber [30], using the specific methodology described below.

Toxicity was measured at three exposure durations so TDT values were calculated for each time series: 15–30-min, 30–45-min and 15–45-min using the appropriate time factor [22]. Time factors were calculated using the following equation:

$$\left(t_{2}-t_{1}\right)/t_{2}$$

with t_2_ being the later timepoint and t_1_ the earlier timepoint. So, the time factors for the 15–30 min, 30–45 min, and 15–45 min time series were 0.5, 0.333, and 0.667, respectively.

The following set of equations was then used to calculate TDT:

$$d=EC_{x_{t_{1}}}-EC_{x_{t_{2}}}$$

$$e=d÷\left(EC_{x_{t_{1}}}\times f_{t_{1}:t_{2}}\right)$$

TDT=e×100

in which EC_x_ is the effect level (i.e. 25%, 50%, 75%), t_2_ is the later time within the exposure time series, t_1_ is the earlier time of that time series, and $f_{t_{1}:t_{2}}$is the appropriate time factor (see above) for the time series. Exemplifying the calculation protocol using the 50% effect level for the 15–45 min time series, the steps were: (a) subtraction of the 45-min EC_50_ from the 15-min EC_50_; (b) dividing that difference by the product of the 15-min EC_50_ value and 0.667; and (c) multiplying that quotient by 100 to put it on a percentage basis. So, for a hypothetical chemical with a 15-min EC_50_ of 15 mg/L and a 45-min EC_50_ of 5 mg/L, the TDT at 50% effect for the 15–45 time series was 100%, as shown below [28]:

a)  15mg/L−5mg/L=10mg/L

b)  10mg/L÷(15mg/L×0.667)=1

c)  1×100=100%

Values for TDT_25_ and TDT_75_ were calculated similarly using the appropriate time factors and EC_25_ or EC_75_ data, respectively.

The only TDT values reported herein are the mean TDT values for the 15–45 min time series (i.e., mean TDT_15-45_). They were calculated separately for each single chemical and mixture by summing the TDT_15-45_ values at the 25%, 50% and 75% effect levels and taking the average [28]. For each experiment, mean TDT_15-45_ values for A and B were then totaled and the average was determined in order to obtain a predicted mixture mean TDT_15-45_ value. For simplicity, from this point on TDT is used as the designation for mean TDT_15-45_.

### 2.5 Calculation of predicted dose-addition and independence curves

The predicted x/y curve for dose-addition was calculated following the procedure described previously [24]. In essence, when agents A and B are dose-additive the EC_50_ for A + B is graphically left-shifted by a dose-ratio (DR) factor of 2 when the agents are equieffective. This is calculated using the equation:

$$Add50=\frac{a50}{DR50}$$

in which Add50 is the EC_50_ for dose-addition, a50 is the EC_50_ of the more potent chemical and b50 (noted below) is the EC_50_ of the less potent agent. The DR50 was determined by:

$$DR50=1+\left(\frac{a50}{b50}\right)$$

Calculation of EC_25_ and EC_75_ values for the predicted dose-addition curve was conducted likewise. Taken together, these predicted values (EC_25_, EC_50_, and EC_75_) and the maximum effect (always 100% herein) permitted calculation of the predicted dose-addition curve via the curve-fitting procedures described above.

Predicted curves for the independence model [31] were calculated using a user-generated transform within SigmaPlot^®^ as:

$$yA+\left[yB\times \frac{\left(100-yA\right)}{100}\right]$$

in which yA and yB are the percent effect values for chemicals A and B, respectively. Independent action is a mixture toxicity concept used to describe, for example, the relative effect of A in the presence of B being equal to the effect of A alone [31], [32].

### 2.6 Combined effect determination

For each mixture, dose-addition and independence quotient values were calculated as measures of combined effect against the dose-addition and independence models. Dose-addition (AQ) and independence (IQ) quotient values at the 25%, 50% and 75% effect levels at a given timepoint were determined by dividing the respective EC_x_ value for the mixture by the predicted EC_x_ value for dose-addition or independence [26]. For example, for a given mixture with a 45-min EC_50_ of 2.42 mg/L, a predicted dose-addition 45-min EC_50_ value of 2.26 mg/L yields an AQ value of 1.07, and a predicted independence 45-min EC_50_ value of 1.87 mg/L yields an IQ value of 1.29. Mixture toxicity has been historically characterized in this lab as being consistent with dose-addition or independence when the respective AQ or IQ value is within the range from 0.90 and 1.10 [26].

Owing to the possibility of the slope of a mixture x/y curve being somewhat different than that for chemical A, chemical B or both chemicals, AQ or IQ values at a given timepoint can, on occasion, be misleading. For example, a mixture might have AQ values of 0.94 at its EC_25_, 1.07 at its EC_50_ and 1.28 at its EC_75_, indicative of the slope difference between the actual mixture x/y curve and the predicted x/y curve for dose-addition. Therefore, two additional metrics: MX/DA and MX/I were developed to more fully assess the combined effect between the 25% and 75% effect levels. To calculate MX/DA, the concentrations of chemical A and concentration equivalents of chemical B within the mixture (i.e., MX) at the EC_25_, EC_50_, and EC_75_ were summed and divided by the sum of the EC_25_, EC_50_, and EC_75_ concentrations for the predicted dose-addition (DA) curve. The MX/I values were calculated in the same manner using the summed EC_25_, EC_50_, and EC_75_ concentrations from the predicted independence (I) curve as the divisor.

## 3 Results

### 3.1 Quality of curve-fitting

In using the 5-parameter logistic minus 1-parameter (5PL-1P) function for fitting all x/y data presented herein, the maximum effect constraint was set to equal 100% (MAX = 100). This was done to provide a consistent basis for determining TDT values and the combined effect. In previous studies using 5PL-1P curve-fitting [26], [27], [28] the maximum effect constraint was set to <100% (MAX < 100). To evaluate the effect this small adjustment had on x/y data fitting, coefficient of determination (r^2^) values were compared (i.e., MAX = 100 vs. MAX < 100) for the entire data set (Table 2). When all r^2^ values were compared the mean and standard deviation were 0.9982 ± 0.0018 for both MAX = 100 and MAX < 100. Since the r^2^ data not was normally distributed (r^2^ values could fall much further below the mean than they could rise above it) the Mann-Whitney rank sum test was used to evaluate median values, with *p* = 0.507 indicating lack of statistical significance between MAX = 100 and MAX < 100-derived r^2^ values.

**Table 2:** Comparison of coefficient of determination (r^2^ ± S.D.) values by maximum effect constraint.

Maximum Effect Constraint	Chem. A 15-min	Chem. B 15-min	Mixture 15-min	Chem. A 30-min	Chem. B 30-min	Mixture 30-min	Chem. A 45-min	Chem. B 45-min	Mixture 45-min
MAX = 100^a^	0.9980 ± 1.5e^−3^	0.9975 ± 2.3e^−3^	0.9980 ± 1.4e^−3^	0.9989 ± 8.1e^−4^	0.9976 ± 2.5e^−3^	0.9984 ± 1.3e^−3^	0.9991 ± 8.2e^−4^	0.9977 ± 2.5e^−3^	0.9987 ± 1.0e^−3^

MAX < 100^b^	0.9981 ± 1.4e^−3^	0.9975 ± 2.3e^−3^	0.9981 ± 1.4e^−3^	0.9989 ± 8.0e^−4^	0.9975 ± 2.6e^−3^	0.9984 ± 1.3e^−3^	0.9991 ± 8.2e^−4^	0.9978 ± 2.5e^−3^	0.9987 ± 1.0e^−3^

a MAX = 100–maximum effect in curve-fitting was set to =100%.

b MAX < 100–maximum effect in curve-fitting was set to be <100%.

Mean r^2^ values for chemicals A, B and the mixtures were compared between the two constraints at each timepoint and were either the same to four decimal places or differed by 0.0001 (Table 2). As evaluated by the *t*-test within each appropriate treatment group (i.e., within each data column of Table 2) there were no statistically significant differences between MAX = 100 and MAX < 100-derived r^2^ values.

### 3.2 Consistency of EC_50_ values across multiple tests of each chemical alone

Calculated EC_50_ values for each chemical tested alone are provided in mg/L for each combination (Table 3). Test-to-test consistency of these values was assessed by determining the coefficient of variation (CV) for each chemical (Table 4). These were typically <20 and always <50.

**Table 3:** EC_50_ values (mg/L) for each chemical tested alone within each combination.

Combination A:B	A: 15-min EC_50_	A: 30-min EC_50_	A: 45-min EC_50_	B: 15-min EC_50_	B: 30-min EC_50_	B: 45-min EC_50_
IAN:IAN	3.01	1.48	0.96	2.93	1.47	0.95
BRAN:BRAN	2.92	1.39	0.87	2.91	1.38	0.85
CLAN:CLAN	157.75	73.84	44.33	156.31	69.96	42.32
CLAN:CLAN	160.78	75.29	45.69	151.78	70.10	42.18

IAN:BRAN	2.93	1.43	0.93	2.80	1.33	0.83
IAN:CLAN	3.12	1.53	0.98	168.11	77.98	47.60
BRAN:CLAN	2.76	1.31	0.81	158.09	74.25	45.28

IAN:3M2B	2.90	1.47	0.95	37.27	40.36	41.51
BRAN:3M2B	2.89	1.37	0.87	40.23	41.33	43.00
CLAN:3M2B	155.97	72.36	45.02	47.59	46.12	43.18

IAN:4NBB	3.17	1.52	0.99	0.62	0.36	0.23
BRAN:4NBB	2.76	1.33	0.86	0.73	0.40	0.27
CLAN:4NBB	146.32	69.77	43.56	0.26	0.13	0.09

IAN:BGE	3.37	1.67	1.15	541.30	594.34	634.83
BRAN:BGE	2.98	1.48	0.97	542.38	576.95	597.36
CLAN:BGE	151.96	72.95	46.26	583.80	588.03	625.25

IAN:DBRAN	2.55	1.22	0.78	1.92	2.14	2.12
BRAN:DBRAN	2.69	1.24	0.77	2.35	2.37	2.30
CLAN:DBRAN	154.33	70.93	43.92	2.47	2.49	2.41

IAN:DCLAN	2.81	1.37	0.89	29.58	19.69	15.39
BRAN:DCLAN	2.73	1.26	0.79	25.23	18.97	15.96
CLAN:DCLAN	176.02	76.48	44.28	24.65	18.29	14.64

IAN:DEM	2.40	1.16	0.75	46.63	37.86	33.38
BRAN:DEM	1.82	0.85	0.52	43.99	38.35	32.92
CLAN:DEM	128.56	64.78	39.11	43.52	36.80	31.49

IAN:EA	3.76	1.81	1.13	122.61	94.82	82.14
BRAN:EA	3.61	1.68	1.02	139.75	108.86	88.24
CLAN:EA	160.25	71.95	46.93	165.26	125.76	109.15

IAN:EAC	3.07	1.51	1.03	1026.47	1113.33	1175.18
BRAN:EAC	2.57	1.24	0.79	1968.51	2070.82	2128.32
CLAN:EAC	156.55	71.24	43.67	1400.62	1486.42	1684.55

IAN:EBAC	3.25	1.63	1.08	1.05	0.41	0.23
BRAN:EBAC	2.90	1.33	0.83	1.01	0.39	0.21
CLAN:EBAC	151.33	69.24	43.74	1.26	0.52	0.29

IAN:ECAC	2.78	1.41	0.91	87.44	55.79	39.40
BRAN:ECAC	2.86	1.34	0.81	96.36	57.58	38.36
CLAN:ECAC	170.79	76.87	46.80	95.48	64.62	41.70

IAN:EFAC	3.53	1.64	1.01	1422.28	1283.86	1313.91
BRAN:EFAC	2.85	1.32	0.82	1305.89	1289.64	1290.91
CLAN:EFAC	168.52	73.61	44.65	1346.58	1283.17	1375.03

IAN:EIAC	2.89	1.41	0.94	0.30	0.12	0.07
BRAN:EIAC	2.74	1.31	0.82	0.28	0.11	0.07
CLAN:EIAC	159.12	73.66	45.70	0.31	0.13	0.08

IAN:EP	3.03	1.43	0.94	2.14	1.05	0.66
BRAN:EP	2.67	1.27	0.79	2.96	1.37	0.84
CLAN:EP	155.56	74.60	46.73	2.06	1.02	0.63

IAN:LIN	2.28	1.17	0.79	16.46	17.32	19.15
BRAN:LIN	2.97	1.49	0.95	16.62	19.14	21.88
CLAN:LIN	146.05	71.05	47.52	13.89	14.01	15.75

IAN:M2BP	2.97	1.57	1.09	38.86	26.49	20.45
BRAN:M2BP	2.77	1.31	0.81	28.00	19.50	15.21
CLAN:M2BP	142.73	67.16	43.44	41.51	27.97	22.79

IAN:MC	3.10	1.58	1.09	204.56	197.93	208.86
BRAN:MC	2.68	1.34	0.86	207.38	170.52	156.11
CLAN:MC	150.90	73.03	44.83	217.51	184.69	188.32

IAN:MVK	3.20	1.70	1.17	0.59	0.33	0.24
BRAN:MVK	2.72	1.30	0.81	0.66	0.34	0.23
CLAN:MVK	155.28	74.25	49.24	0.72	0.39	0.28

IAN:NER	3.06	1.57	1.07	8.89	10.07	11.30
BRAN:NER	3.10	1.50	1.00	6.80	7.60	8.28
CLAN:NER	167.31	80.96	52.75	8.72	9.41	10.48

IAN:PN	3.03	1.47	0.96	3926.46	3628.92	3269.04
BRAN:PN	2.81	1.32	0.84	3742.01	3354.16	2972.09
CLAN:PN	154.59	74.19	45.40	4026.94	3794.69	3400.06

IAN:TCLAN	2.95	1.41	0.93	1.22	0.94	0.90
BRAN:TCLAN	2.73	1.32	0.82	1.62	1.20	1.00
CLAN:TCLAN	155.21	70.98	44.47	1.11	0.90	0.82

**Table 4:** CV^a^ values for EC_50_ data.

Chemical (n)	15-min	30-min	45-min
IAN (24)	10.7	10.6	11.4
BRAN (24)	10.4	10.7	11.4
CLAN (26)	6.2	4.7	5.6
3M2B (3)	12.7	7.2	2.2
4NBB (3)	45.8	49.1	48.0
BGE (3)	4.4	1.5	3.1
DBRAN (3)	12.9	7.6	6.4
DCLAN (3)	10.2	3.7	4.3
DEM (3)	3.7	2.1	3.0
EA (3)	15.1	14.1	15.2
EAC (3)	32.4	31.0	28.7
EBAC (3)	12.1	15.9	17.1
ECAC (3)	5.3	7.9	4.3
EFAC (3)	2.5	0.3	3.3
EIAC (3)	5.2	8.3	7.9
EP (3)	20.9	16.9	16.0
LIN (3)	9.8	15.5	16.2
M2BP (3)	19.8	18.4	19.9
MC (3)	3.2	7.4	14.4
MVK (3)	9.9	9.1	10.6
NER (3)	14.3	14.2	15.6
PN (3)	3.7	6.2	6.8
TCLAN (3)	20.4	14.8	9.9

a Coefficient of variation.

### 3.3 Combined effects

**Fig. 1:**
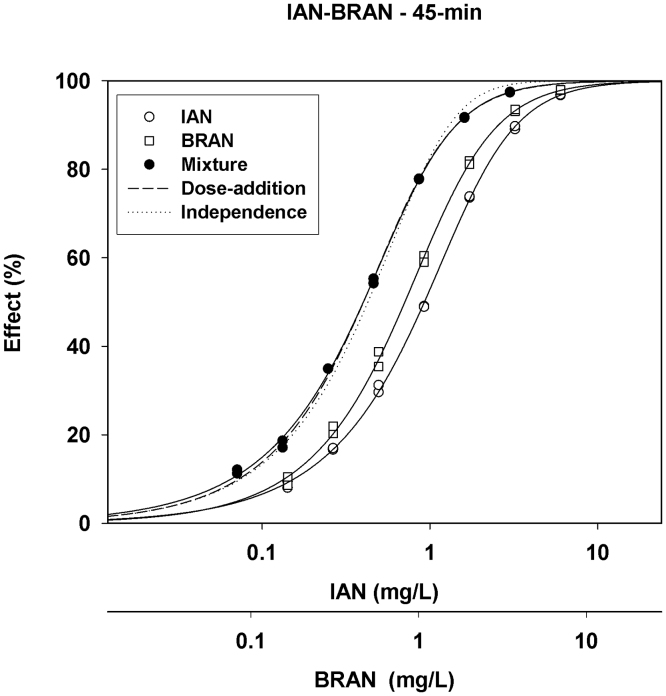
Concentration-effect curves for iodoacetonitrile (IAN), bromoacetonitrile (BRAN) and the IAN-BRAN mixture after 45-min of exposure, along with predicted curves for dose-addition and independence. Mixture toxicity was consistent with both combined effects models. The predicted dose-addition curve is almost completely covered by the mixture curve above about 35% effect.

Combined effects of mono-halogenated acetonitrile-containing mixtures versus the dose-addition (Table 5) and independence (Table 6) models varied depending on the chemical combination being examined and were generally, but not always, consistent between the EC_50_-AQ and MX/DA metrics and between the EC_50_-IQ and MX/I metrics. Sham (e.g., IAN-IAN) and true combinations of mono-halogenated acetonitriles (e.g., IAN-BRAN) were consistent with both dose-addition and independence as the predicted curves for the two combined effect models tend to overlap for these chemicals (e.g., Fig. 1).

**Table 5:** Mixture toxicity versus dose-addition using the AQ^a^ and MX/DA^b^ metrics.

Combination	15-min EC_50_ AQ	MX/DA	30-min EC_50_ AQ	MX/DA	45-min EC_50_ AQ	MX/DA
IAN-IAN	**0.99**^c^	**1.00**	**1.01**	**1.01**	**1.03**	**1.03**
BRAN-BRAN	**1.00**	**1.01**	**1.01**	**1.01**	**1.06**	**1.05**
CLAN-CLAN	**0.99**	**1.00**	**0.99**	**1.00**	**1.01**	**1.01**
CLAN-CLAN	**0.96**	**0.96**	**0.95**	**0.95**	**0.98**	**0.97**

IAN-BRAN	**1.00**	**1.00**	**1.01**	**1.01**	**1.00**	**1.00**
IAN-CLAN	**0.96**	**0.96**	**0.96**	**0.96**	**0.99**	**0.99**
BRAN-CLAN	**1.04**	**1.02**	**1.04**	**1.03**	**1.05**	**1.04**

IAN-3M2B	**1.10**	**1.10**	1.11^d^	1.16	1.22	1.27
BRAN-3M2B	**1.10**	**1.08**	1.17	1.21	1.22	1.32
CLAN-3M2B	**1.07**	1.20	1.20	1.34	1.36	1.51

IAN-4NBB	1.22	1.26	**1.04**	**1.07**	**0.97**	**0.97**
BRAN-4NBB	1.28	1.43	1.28	1.32	1.27	1.31
CLAN-4NBB	1.20	1.22	1.24	1.22	1.20	1.17

IAN-BGE	1.18	1.17	1.28	1.28	1.35	1.37
BRAN-BGE	1.14	1.19	1.32	1.38	1.46	1.51
CLAN-BGE	1.13	1.21	1.32	1.38	1.41	1.46

IAN-DBRAN	1.42	1.48	1.28	1.34	1.21	1.28
BRAN-DBRAN	1.22	1.22	1.26	1.28	1.23	1.26
CLAN-DBRAN	1.15	1.14	1.22	1.22	1.19	1.19

IAN-DCLAN	1.17	1.27	1.19	1.29	1.18	1.28
BRAN-DCLAN	1.13	1.20	1.16	1.30	1.21	1.38
CLAN-DCLAN	1.25	1.39	1.15	1.33	1.14	1.31

IAN-DEM	**1.09**	1.13	1.15	1.23	**1.08**	1.16
BRAN-DEM	**1.10**	1.18	1.11	1.22	1.15	1.27
CLAN-DEM	1.24	1.35	1.21	1.31	1.24	1.34

IAN-EA	1.19	1.24	1.16	1.22	1.23	1.28
BRAN-EA	1.20	1.24	1.20	1.27	1.21	1.27
CLAN-EA	1.19	1.26	1.19	1.25	1.20	1.26

IAN-EAC	1.17	1.12	1.28	1.21	1.26	1.23
BRAN-EAC	**1.02**	**1.04**	1.21	1.21	1.37	1.36
CLAN-EAC	**1.10**	**1.07**	1.21	1.20	1.39	1.36

IAN-EBAC	**0.97**	**0.98**	**0.94**	**0.95**	**0.91**	**0.92**
BRAN-EBAC	**1.01**	**1.00**	**0.97**	**0.97**	**0.96**	**0.96**
CLAN-EBAC	**0.97**	**0.96**	**0.94**	**0.95**	**0.93**	**0.94**

IAN-ECAC	1.12	1.24	**1.03**	1.11	**0.95**	**1.02**
BRAN-ECAC	1.13	1.25	1.12	1.19	**1.06**	1.11
CLAN-ECAC	**1.10**	1.23	**1.05**	1.11	**1.03**	**1.09**

IAN-EFAC	*0.84*^e^	**0.92**	**0.93**	**1.02**	**0.93**	**1.02**
BRAN-EFAC	**0.98**	**0.99**	**0.91**	**0.95**	**0.93**	**0.97**
CLAN-EFAC	**0.95**	**0.94**	**0.90**	**0.91**	**0.91**	**0.93**

IAN-EIAC	**0.95**	**0.96**	**0.96**	**0.96**	**0.96**	**0.95**
BRAN-EIAC	**0.98**	**0.98**	**0.96**	**0.97**	**0.93**	**0.94**
CLAN-EIAC	**0.95**	**0.92**	**0.97**	**0.96**	**0.96**	**0.96**

IAN-EP	**1.08**	**1.09**	**1.10**	**1.10**	**1.08**	**1.09**
BRAN-EP	*0.87*	*0.86*	*0.88*	*0.89*	**0.91**	**0.92**
CLAN-EP	**0.92**	**0.93**	**0.93**	**0.95**	**0.93**	**0.94**

IAN-LIN	1.11	1.18	**1.07**	1.18	**1.09**	1.21
BRAN-LIN	**0.97**	**1.06**	**0.98**	**1.10**	**0.99**	1.14
CLAN-LIN	**0.93**	1.11	**1.02**	1.17	**1.01**	1.18

IAN-M2BP	1.25	1.31	1.30	1.34	1.28	1.31
BRAN-M2BP	1.22	1.26	1.26	1.31	1.26	1.30
CLAN-M2BP	1.40	1.51	1.46	1.52	1.37	1.43

IAN-MC	**1.09**	1.16	1.18	1.24	1.27	1.31
BRAN-MC	1.13	1.15	1.15	1.17	1.20	1.23
CLAN-MC	1.15	1.21	1.27	1.28	1.39	1.39

IAN-MVK	**1.05**	**1.06**	**1.05**	**1.05**	**1.03**	**1.03**
BRAN-MVK	**0.96**	**0.93**	**0.99**	**0.97**	**1.04**	**1.02**
CLAN-MVK	**1.02**	**0.97**	**1.00**	**0.99**	**0.98**	**0.98**

IAN-NER	**1.06**	1.13	1.14	1.21	1.20	1.27
BRAN-NER	1.13	1.16	1.20	1.25	1.29	1.36
CLAN-NER	1.14	1.20	1.24	1.29	1.29	1.36

IAN-PN	1.19	1.17	1.24	1.24	1.25	1.26
BRAN-PN	1.29	1.26	1.33	1.33	1.30	1.33
CLAN-PN	1.33	1.32	1.35	1.36	1.40	1.40

IAN-TCLAN	1.57	1.48	1.48	1.37	1.39	1.31
BRAN-TCLAN	1.19	1.15	1.16	1.12	1.20	1.15
CLAN-TCLAN	1.30	1.23	1.19	1.15	1.17	1.14

a AQ – EC_50_ additivity quotient for dose-addition – see text for calculation procedures.

b MX/DA – Mixture/dose-addition – see text for calculation procedures.

c Bolded text – combined effect consistent with dose-addition.

d Normal text – combined effect less-than that predicted by dose-addition.

e Italicized text – combined effect greater-than that predicted by dose-addition.

**Table 6:** Mixture toxicity versus independence using the IQ^a^ and MX/I^b^ metrics.

Combination	15-min EC_50_ IQ	MX/I	30-min EC_50_ IQ	MX/I	45-min EC_50_ IQ	MX/I
IAN-IAN	**0.90**^c^	**0.96**	**0.92**	**0.95**	**0.95**	**0.97**
BRAN-BRAN	**0.94**	**0.98**	**0.95**	**0.97**	**0.99**	**1.01**
CLAN-CLAN	**0.92**	**0.96**	**0.94**	**0.98**	**0.97**	**1.00**
CLAN-CLAN	**0.90**	**0.93**	**0.90**	**0.92**	**0.93**	**0.94**

IAN-BRAN	**0.91**	**0.95**	**0.93**	**0.96**	**0.94**	**0.96**
IAN-CLAN	*0.87*^d^	**0.92**	*0.88*	**0.91**	**0.92**	**0.94**
BRAN-CLAN	**0.96**	**0.99**	**0.96**	**0.99**	**0.97**	**1.00**

IAN-3M2B	**1.07**	1.21^e^	1.13	1.30	1.28	1.46
BRAN-3M2B	**1.09**	1.23	1.21	1.40	1.28	1.50
CLAN-3M2B	**1.10**	1.37	1.27	1.54	1.47	1.74

IAN-4NBB	1.21	1.45	**1.06**	1.23	**1.01**	1.13
BRAN-4NBB	1.43	1.75	1.41	1.62	1.37	1.56
CLAN-4NBB	1.21	1.37	1.30	1.41	1.26	1.35

IAN-BGE	1.26	1.40	1.30	1.44	1.35	1.48
BRAN-BGE	1.24	1.39	1.45	1.60	1.56	1.69
CLAN-BGE	1.28	1.48	1.50	1.67	1.51	1.67

IAN-DBRAN	1.17	1.24	**1.06**	1.11	**1.04**	**1.09**
BRAN-DBRAN	**1.07**	**1.06**	1.16	1.15	1.18	1.19
CLAN-DBRAN	**0.98**	**1.07**	**1.08**	**1.06**	**1.06**	**1.05**

IAN-DCLAN	1.22	1.45	1.26	1.50	1.27	1.47
BRAN-DCLAN	1.11	1.31	1.19	1.47	1.27	1.55
CLAN-DCLAN	1.35	1.63	1.34	1.66	1.34	1.66
						
IAN-DEM	**1.07**	1.21	1.16	1.33	1.12	1.26
BRAN-DEM	**1.07**	1.25	**1.08**	1.28	1.11	1.31
CLAN-DEM	1.26	1.48	1.21	1.41	1.26	1.45

IAN-EA	1.22	1.33	1.18	1.28	1.22	1.31
BRAN-EA	1.20	1.33	1.21	1.34	1.20	1.32
CLAN-EA	1.31	1.42	1.34	1.44	1.34	1.42

IAN-EAC	1.20	1.29	1.38	1.41	1.38	1.42
BRAN-EAC	1.12	1.23	1.33	1.42	1.47	1.55
CLAN-EAC	1.19	1.31	1.31	1.43	1.49	1.57

IAN-EBAC	**0.96**	**1.02**	**0.97**	**1.00**	**0.98**	**0.98**
BRAN-EBAC	**1.02**	**1.05**	**1.07**	**1.08**	**1.08**	**1.08**
CLAN-EBAC	**0.98**	**1.02**	**1.02**	**1.03**	**1.07**	**1.05**

IAN-ECAC	1.18	1.46	**1.10**	1.27	**1.04**	1.17
BRAN-ECAC	1.20	1.47	1.20	1.39	1.17	1.31
CLAN-ECAC	1.20	1.47	1.13	1.29	1.14	1.28

IAN-EFAC	**0.93**	1.18	**1.10**	1.32	1.16	1.30
BRAN-EFAC	1.15	1.31	**1.08**	1.21	1.18	1.24
CLAN-EFAC	**1.06**	1.21	**1.10**	1.19	1.15	1.19

IAN-EIAC	**0.94**	**1.00**	**0.98**	**1.01**	**1.02**	**1.03**
BRAN-EIAC	**0.98**	**1.03**	**1.01**	**1.04**	**0.98**	**1.01**
CLAN-EIAC	**0.93**	**0.95**	**0.97**	**0.99**	**0.99**	**1.02**

IAN-EP	1.16	1.21	1.15	1.20	1.14	1.18
BRAN-EP	*0.83*	*0.87*	*0.85*	*0.89*	**0.90**	**0.94**
CLAN-EP	**0.92**	**0.97**	**0.94**	**0.99**	**0.98**	**1.01**

IAN-LIN	1.21	1.44	1.19	1.45	1.19	1.44
BRAN-LIN	1.11	1.31	1.14	1.38	1.18	1.43
CLAN-LIN	**1.00**	1.39	1.18	1.50	1.15	1.47

IAN-M2BP	1.24	1.37	1.29	1.40	1.29	1.38
BRAN-M2BP	1.15	1.24	1.18	1.25	1.19	1.26
CLAN-M2BP	1.36	1.56	1.46	1.60	1.35	1.48

IAN-MC	**1.10**	1.25	1.19	1.29	1.21	1.28
BRAN-MC	**1.10**	1.18	1.11	1.18	1.16	1.22
CLAN-MC	1.25	1.37	1.38	1.44	1.44	1.49

IAN-MVK	**1.09**	1.19	**1.10**	1.15	**1.06**	**1.10**
BRAN-MVK	**0.93**	**0.97**	**0.94**	**0.98**	**0.99**	**1.02**
CLAN-MVK	**1.08**	1.11	**1.08**	1.12	**1.04**	**1.08**

IAN-NER	1.23	1.36	1.23	1.38	1.25	1.38
BRAN-NER	1.18	1.30	1.25	1.40	1.33	1.49
CLAN-NER	1.24	1.39	1.34	1.49	1.35	1.52

IAN-PN	1.12	1.13	1.17	1.18	1.19	1.20
BRAN-PN	1.22	1.21	1.28	1.28	1.25	1.28
CLAN-PN	1.28	1.29	1.27	1.30	1.33	1.34

IAN-TCLAN	1.68	1.76	1.60	1.64	1.50	1.55
BRAN-TCLAN	1.14	1.22	1.12	1.18	1.19	1.22
CLAN-TCLAN	1.28	1.33	1.17	1.24	1.15	1.20

a IQ – EC_50_ independence quotient – see text for calculation procedures.

b MX/I – Mixture/independence – see text for calculation procedures.

c Bolded text – combined effect consistent with independence.

d Italicized text – combined effect greater-than that predicted by independence.

e Normal text – combined effect less-than that predicted by independence.

**Fig. 2:**
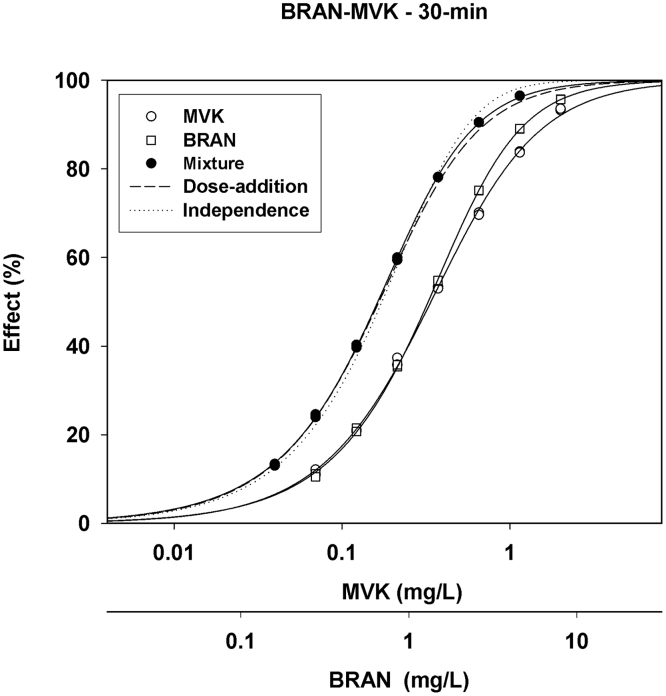
Concentration-effect curves for bromoacetonitrile (BRAN), methyl vinyl ketone (MVK) and the BRAN-MVK mixture after 30-min of exposure, along with predicted curves for dose-addition and independence. Mixture toxicity was consistent with both combined effects models.

Several other chemicals tested with the mono-halogenated acetonitriles also produced combined effects that were predominantly consistent with dose-addition (Table 5), such as ethyl bromoacetate (EBAC), ethyl fluoroacetate (EFAC), ethyl iodoacetate (EIAC), and methyl vinyl ketone (MVK; e.g., with BRAN, Fig. 2). For ethyl propiolate (EP) the combined effect with IAN and CLAN was consistent with dose addition across all three timepoints but when EP was tested with BRAN, slightly greater-than dose-additive toxicity was observed at 15 and 30-min of exposure before becoming consistent with dose-addition at 45-min. For ethyl chloroacetate (ECAC) the combined effect with the XANs (i.e., X = I, Br or Cl) varied somewhat from dose-addition, especially at 15 and 30-min. Two of the agents (EBAC, EIAC) produced combined effects that were also consistent with independence across the three timepoints when given with each XAN. A third chemical (MVK) also produced some instances of consistency with independence when given with each XAN, but there were also instances of the mixture toxicity being less-than predicted by the independence model at 15 and 30-min (Table 6).

For most of the remaining combinations, with specific exceptions at individual timepoints, mixture toxicity was less-than that predicted by both the dose-addition and independence models. These results tended to be consistent between the EC_50_-AQ and MX/DA metrics for dose-addition (Table 5) and between the EC_50_-IQ and MX/I metrics for independence (Table 6). Again a metric value > 1.10 was used to indicate a combined effect less-than that predicted by the model. One prominent exception to this consistency was noted when each of the XANs was tested with linalool (LIN). At the 45-min timepoint, the EC_50_-AQ values suggested a dose-additive combined effect for IAN-LIN, BRAN-LIN and CLAN-LIN, in contrast to the MX/DA metric values which indicated a less-than dose-additive combined effect.

A few instances of combined effects greater-than predicted by the models were also observed. Although at the early and middle timepoints two combinations showed greater-than dose-additive toxicity (Table 5: IAN-EFAC – at 15-min for only the EC_50_-AQ metric; BRAN-EP at 15 and 30-min for both metrics) and two combinations showed greater-than independent effects (Table 6: IAN-CLAN at 15 and 30-min for only the EC50-IQ metric; BRAN-EP at 15 and 30-min for both metrics), enhanced mixture toxicity was not observed at the 45-min timepoint for any of the combinations based on the predicted effects for either metric of each model.

### 3.4 TDT and combined effect

Time-dependent toxicity (TDT) values were always 95% or greater for the mono-halogenated acetonitriles tested singly, while those values for the other chemicals spanned the range from being negative to greater-than 100% (i.e., −46% to 121% – Table 7, Table 8). Using the MX/DA metric for combined effect, Table 7 shows the combinations for which mixture toxicity was consistent with dose-addition after 45-min of exposure. Table 8 shows the combinations for which mixture toxicity via the MX/DA metric was not consistent with dose-addition after 45-min. Please note in these tables that for each combination the TDT values reported are presented as the chemical with the higher individual TDT (column 2) and the one with the lower individual TDT (column 3); they are not necessarily the first- and second-listed chemicals of the combination (column 1). For example, in Table 7 for the BRAN-CLAN combination, CLAN had the higher TDT (107.9) and BRAN the lower TDT (106.1). Also given are the predicted mixture TDT value (TDT_p_) (i.e., the average of the TDT values of the chemicals in the combination – column 4), the observed TDT value (TDT_o_) obtained in testing (column 5), and the difference between the observed TDT and predicted TDT (TDT_o-p_ – column 6). For ease of reference the 45-min MX/DA values are also provided (column 7).

**Table 7:** Time-dependent toxicity (TDT) values for dose-additive combinations.

Combination	Higher^a^ TDT	Lower^b^ TDT	TDT^c^ Predicted	TDT^d^ Observed	TDT^e^ Obs. – Pred.	MX/DA^f^ (45-min)
BRAN-BRAN	106.7	105.6	106.2	104.2	−2.0	**1.05**^g^
BRAN-CLAN	107.9	106.1	107.0	106.8	−0.2	**1.05**
BRAN-EBAC	120.6	107.5	114.1	117.3	3.2	**0.96**
BRAN-EFAC	107.0	**19.9**	63.5	79.0	**15.5**	**0.97**
BRAN-EIAC	115.4	104.8	110.1	113.5	3.4	**0.94**
BRAN-EP	109.5	106.3	107.7	104.4	−3.3	**0.92**
BRAN-MVK	106.1	99.4	102.8	99.7	−3.1	**1.02**
CLAN-CLAN	109.7	109.2	109.5	108.6	−0.9	**1.01**
CLAN-CLAN	107.9	107.7	107.8	107.5	−0.3	**0.97**
CLAN-EBAC	118.3	107.6	113.0	114.5	1.5	**0.94**
CLAN-ECAC	108.8	88.1	98.5	101.8	3.3	**1.09**
CLAN-EFAC	111.0	**18.9**	65.0	82.6	**17.6**	**0.93**
CLAN-EIAC	115.9	107.3	111.6	112.6	1.0	**0.96**
CLAN-EP	106.1	105.9	106.0	104.5	−1.5	**0.94**
CLAN-MVK	103.5	90.9	97.2	97.6	0.4	**0.98**
IAN-4NBB	105.1	95.6	100.3	108.7	8.4	**0.97**
IAN-BRAN	105.7	103.8	104.8	104.4	−0.4	**1.00**
IAN-CLAN	108.1	103.7	105.9	104.4	−1.5	**0.99**
IAN-EBAC	119.1	101.9	110.5	116.4	5.9	**0.92**
IAN-ECAC	101.8	86.1	94.0	101.8	7.8	**1.02**
IAN-EFAC	107.9	28.6	68.3	67.5	−0.8	**1.02**
IAN-EIAC	118.0	102.6	110.3	113.2	2.9	**0.95**
IAN-EP	104.3	102.7	103.5	103.1	−0.4	**1.09**
IAN-IAN	103.2	102.9	103.1	101.6	−1.5	**1.03**
IAN-MVK	96.0	90.6	93.3	93.7	0.4	**1.03**

a Individual chemical having the higher TDT value in the combination.

b Individual chemical having the lower TDT value in the combination.

c Predicted TDT for dose-addition = average of the higher and lower TDT values.

d TDT of the mixture obtained in testing.

e Observed TDT minus predicted TDT.

f Combined effect metric for dose addition at 45-min of exposure – see text for calculation procedures.

g Bolded text used for emphasis.

**Table 8:** Time-dependent toxicity (TDT) values for non-dose-additive combinations.

Combination	Higher^a^ TDT	Lower^b^ TDT	TDT^c^ Predicted	TDT^d^ Observed	TDT^e^ Obs. – Pred.	MX/DA^f^ (45-min)
BRAN-3M2B	106.0	−10.2	47.9	40.3	−7.6	1.32
BRAN-4NBB	103.0	93.5	98.3	102.6	4.3	1.31
BRAN-BGE	100.5	−11.3	44.6	39.4	−5.2	1.51
BRAN-DBRAN	106.9	**5.5**^g^	56.2	77.7	**21.2**	1.26
BRAN-DCLAN	107.4	57.9	82.7	76.4	−6.3	1.38
BRAN-DEM	107.4	40.6	74.0	79.2	5.2	1.27
BRAN-EA	108.9	55.4	82.2	81.7	−0.5	1.27
BRAN-EAC	103.6	−17.9	42.9	48.6	5.7	1.36
BRAN-ECAC	107.7	93.0	100.4	103.9	3.5	1.11
BRAN-LIN	101.5	**−43.2**	29.2	53.1	**23.9**	1.14
BRAN-M2BP	106.9	70.5	88.7	85.4	−3.3	1.30
BRAN-MC	102.3	37.7	70.0	69.3	−0.7	1.23
BRAN-NER	100.9	−34.2	33.4	38.2	4.8	1.36
BRAN-PN	106.3	26.6	66.5	68.0	1.5	1.33
BRAN-TCLAN	106.1	58.1	82.1	87.5	5.4	1.15
CLAN-3M2B	107.5	15.6	61.6	55.4	−6.2	1.51
CLAN-4NBB	106.0	100.0	103.0	102.4	−0.6	1.17
CLAN-BGE	103.9	−7.9	48.0	52.1	4.1	1.46
CLAN-DBRAN	107.1	**3.3**	55.2	78.1	**22.9**	1.19
CLAN-DCLAN	112.6	62.5	87.6	91.5	3.9	1.31
CLAN-DEM	104.3	43.6	74.0	77.2	3.2	1.34
CLAN-EA	105.5	53.4	79.5	81.1	1.6	1.26
CLAN-EAC	108.3	−30.0	39.2	40.4	1.2	1.36
CLAN-LIN	102.1	−18.6	41.8	48.7	6.9	1.18
CLAN-M2BP	105.2	68.5	86.9	91.0	4.1	1.43
CLAN-MC	104.8	18.1	61.5	54.1	−7.4	1.39
CLAN-NER	102.5	**−35.5**	33.5	43.9	**10.4**	1.36
CLAN-PN	106.7	19.9	63.3	71.8	8.5	1.40
CLAN-TCLAN	107.2	42.7	75.0	84.2	9.2	1.14
IAN-3M2B	102.6	**−13.8**	44.4	33.4	**−11.0**	1.27
IAN-BGE	98.9	−21.0	39.0	44.3	5.3	1.37
IAN-DBRAN	103.4	**−13.7**	44.9	80.8	**35.9**	1.28
IAN-DCLAN	103.4	73.6	88.5	87.5	−1.0	1.28
IAN-DEM	104.9	45.5	75.2	76.9	1.7	1.16
IAN-EA	105.2	50.2	77.7	71.4	−6.3	1.28
IAN-EAC	100.8	−21.3	39.8	47.9	8.1	1.23
IAN-LIN	97.7	**−24.4**	36.7	60.8	**24.1**	1.21
IAN-M2BP	95.4	72.4	83.9	82.5	−1.4	1.31
IAN-MC	98.1	−6.3	45.9	40.5	−5.4	1.31
IAN-NER	95.7	**−45.7**	25.0	40.9	**15.9**	1.27
IAN-PN	104.6	20.9	62.8	61.9	−0.9	1.26
IAN-TCLAN	103.4	**40.7**	72.1	84.3	**12.2**	1.31

a Individual chemical having the higher TDT value in the combination.

b Individual chemical having the lower TDT value in the combination.

c Predicted TDT for dose-addition = average of the higher and lower TDT values.

d TDT of the mixture obtained in testing.

e Observed TDT minus predicted TDT.

f Combined effect metric for dose addition at 45-min of exposure – see text for calculation procedures.

g Bolded text used for emphasis.

In the study, 25 combinations were consistent with dose-addition by the MX/DA metric after 45-min and the mean ± S.E. for the TDT_o-p_ values of those combinations was 3.5 ± 1.1. Of the 25 dose-additive combinations, 23 (92%) had TDT_o-p_ values that were <10. The combinations with TDT_o-p_ that were >10 contained EFAC, which when tested alone had individual TDT values that were <30%. So, the difference in TDT between the chemical with the higher TDT and the TDT of EFAC was always >60%. All dose-additive combinations that did not contain EFAC had a difference between the chemical with the higher TDT and the chemical with the lower TDT that was >25% (Table 7). Also, of the 25 combinations that were consistent with dose-addition, 22 (88%) had individual TDT values that were >80% for both chemicals.

There were 42 combinations that produced a combined effect that was not consistent with dose-addition after 45-min of exposure when using the MX/DA metric and the mean ± S.E. for the TDT_o-p_ values was 7.6 ± 1.5 (Table 8). Of those 42 combinations, 33 (78.6%) had TDT_o-p_ values that were <10. In the nine cases where TDT_o-p_ values were >10, the TDT value for the chemical with lower TDT was always <45%. Once again, the difference in TDT of the chemical with the higher TDT and that with the lower TDT was always >60%. In contrast to the dose-additive combinations (Table 7), the non-dose-additive combinations (Table 8) had only two instances in which both chemicals had individual TDT values above 80% (i.e., 88% vs. 4.8%, respectively). Overall, however, the TDT_o-p_ values for the non-dose-additive combinations were not statistically different from the values for dose-additive combinations (*t*-test, *p* = 0.291).

## 4 Discussion

Of the 67 combinations for which data appear in this report, results for 28 combinations are presented for the first time. Data for 10 combinations included herein were originally published as having been analyzed using the 4-parameter logistic function under the maximum effect constraint – MAX < 100 [24] and again using the 5PL-1P function also with MAX < 100 [26]. Included in the latter paper were 12 other combinations also included here – but analyzed using the 5PL-1P function with MAX < 100. Also, TDT values for an additional 17 combinations derived using the 5PL-1P function and MAX < 100% were included in the most recent report [28], but their combined effects have not been previously reported. Thus, this report presents for the first time mixture toxicity assessments for 45 combinations and it is the first report for which any x/y data from the 67 combinations were derived using the constraint MAX = 100.

### 4.1 Quality of curve-fitting

To evaluate the quality of fitting for the 5PL-1P function when using MAX = 100, r^2^ values for each single chemical and mixture curve (n = 603) were compiled and compared to those obtained for the 5PL-1P with MAX < 100. The average r^2^ value was the same for both, not surprisingly, since the constraint MAX < 100 often yielded a maximum effect equal to 100%. A comparison was made across all nine x/y curve categories (i.e., A, B, A + B, at each of the three timepoints) and no statistically significant differences were found for r^2^ values calculated using MAX = 100 vs. MAX < 100, suggesting that either constraint can be used for chemicals having toxicity that approaches the top of the x/y curve.

### 4.2 Consistency of EC_50_ values across multiple tests of each chemical alone

Test-to-test consistency of all individual chemical EC_50_ data was evaluated for all 23 chemicals used in testing by determining coefficient of variation (CV) values. This approach is preferred over using standard deviation values when data are generated by multiple operators [33]. The EC_50_ CV values reported are considered acceptable for test-to-test variation [34], [35] and, with the exception of those for 4-nitrobenyl bromide (4NBB), typical of those generated in this lab [26]. The higher EC_50_ CV values for 4NBB appear to be associated with some unique feature of 4NBB toxicity in this model system.

### 4.3 Combined effects

**Fig. 3:**
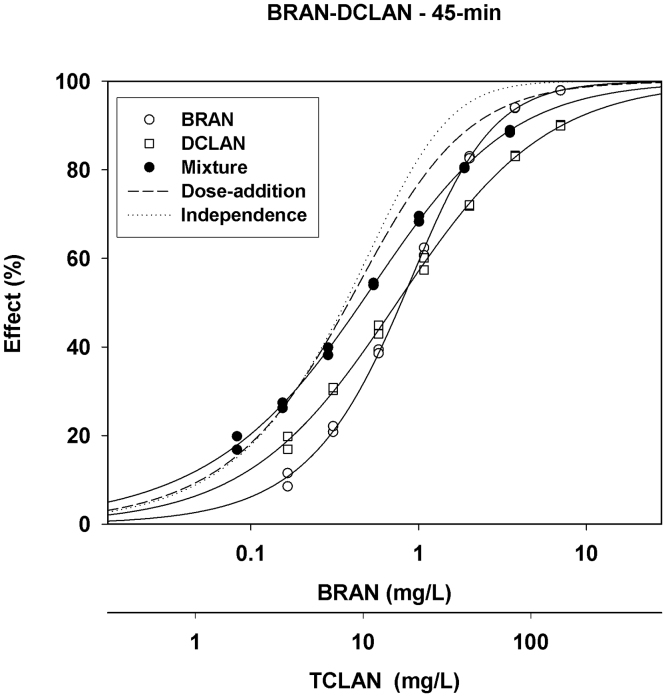
Concentration-effect curves for bromoacetonitrile (BRAN), dichloroacetonitrile (DCLAN) and the BRAN-DCLAN mixture after 45-min of exposure, along with predicted curves for dose-addition and independence. Mixture toxicity was less-than predicted for both combined effects models.

**Fig. 4:**
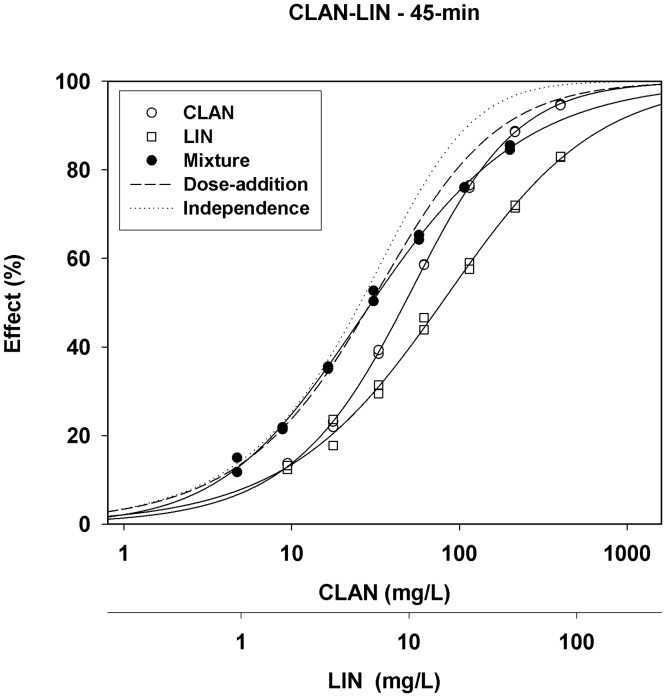
Concentration-effect curves for chloroacetonitrile (CLAN), linalool (LIN) and the CLAN-LIN mixture after 45-min of exposure, along with predicted curves for dose-addition and independence. Mixture toxicity was consistent with the combined effects models at lower effect levels but was right-shifted from those models above about 60% effect.

It was previously reported that both sham and true combinations of XANs produced combined effects consistent with dose-addition and independence when analyzed using the 5PL-1P function [26]. This was also the tendency for combinations of the ethyl α-halogenated acetates with the XANs, with the exception that the chloro and fluoro ethyl acetates had a combined effect typically less-than that expected for independence [26]. For these combinations, the predicted dose-addition and independence curves were very close to overlapping below about the 80% effect level (see Fig. 1, Fig. 2). Even for combinations that produced a combined effect less-than that predicted by either model, the predicted dose-addition and independence curves were close together at lower effect levels (see Fig. 3). This is predominantly due to the chemicals being tested having x/y curve slopes <3. When at least one of the chemicals has a steeper slope of the x/y curve, the predicted dose-addition and independence curves will be more clearly separated [36 – see Figs. 3–5 therein]. Therefore it is not surprising that some combinations resulted in mixture toxicity that was consistent with both models.

It is noted that on occasion the observed mixture toxicity will often be close to the predicted dose-addition (and/or independence) curve at lower effect levels but become gradually more right-shifted from it at higher effect levels (see Fig. 3, Fig. 4), thereby indicating mixture toxicity being less-than that predicted by the model at those points. The MX/DA and MX/I metrics were developed to provide a means of recognizing this phenomenon when x/y data are not shown graphically. These metrics simply total the concentration of chemical A and the relative concentration of chemical B in the mixture at the EC_25_, EC_50_ and EC_75_ and divide them by the sum of the concentrations of the chemicals at the same three effect levels for either the predicted dose-addition curve (i.e., MX/DA) or the predicted independence curve (i.e., MX/I). For the CLAN-LIN combination, the 45-min EC_50_-AQ value was 1.01 but the MX/DA value for the mixture was 1.18. By examining Fig. 4 it can be seen that the observed toxicity tracks well with the predicted dose-addition curve between 10 and 50% effect, but begins to be shifted to the right of the dose-addition curve at higher effect levels. A similar response can be seen for the BRAN-DCLAN (dichloroacetonitrile) combination but with the right-shift beginning at a lower effect level (Fig. 3). While one may argue that it is at the lower effect levels that one would be more likely to encounter chemical concentrations that could be environmentally relevant, the MX/DA metric highlights combinations for which the mixture x/y curve drifts away from the predicted dose-addition curve at some point, being thereby suggestive of a difference in mode of toxic action between the chemicals. This, along with differences in slope values, chemical potencies (i.e., 1/EC_50_) and TDT – have the potential to be useful in predicting chemical mixture toxicity by mode of toxic action; a primary intent of the studies being conducted in this lab.

### 4.4 TDT and combined effect

As noted above (Section 3.4), observed TDT minus predicted TDT (i.e., TDT_o-p_) values were usually <10, thereby being consistent with the previous finding that the average of the TDT values for the individual chemicals in a binary mixture could be used to predict the TDT of the mixture [28]. This conclusion is supported by TDT_o-p_ values for non-dose-additive combinations not being significantly different from those for dose-additive combinations. It was noted, however, that for 22 of 25 combinations that had toxicity consistent with dose-addition via the MX/DA metric both chemicals in the combination had individual TDT values >80%. In contrast, for combinations that were not consistent with dose-addition only 2 of the 42 had both chemicals with individual TDT values >80%. As TDT is explained by irreversible action, chemicals with toxicity that is fully time-dependent (i.e., TDT ≥100%) exert toxic effects that are fully irreversible, while those lacking TDT (i.e., TDT ≤0%) show only reversible toxic effects and those with TDT between 0 and 100% have toxicity that is partly irreversible and partly reversible. For a chemical in the latter group, it is suggested that two (or more) modes of toxicity are being exerted within the range of chemical concentrations used in testing. Since in this study at least one of the chemicals in the mixture always had high TDT (i.e., >95%) further study is needed on combinations for which the chemicals included in testing are limited to those having low (i.e., <20%) and/or middle (i.e., 20%–80%) range TDT values. The results of this study support the conclusion [28] that simply knowing the TDT of the individual chemicals in a mixture could be useful for predicting the combined effect.

## 5 Conclusions

Evaluation of TDT and combined effects for a series of mono-halogenated acetonitrile-containing mixtures suggests that the relative TDT level of the individual components is a factor in whether the toxicity of the mixture is consistent with dose-addition or not. After 45-min of exposure the results showed that when both chemicals of the combination had individual TDT values >80%, toxicity of the mixture was more likely to be consistent with that predicted for dose-addition (i.e., >85% frequency). In contrast when one chemical of the combination had a TDT value >80% and the second chemical had a TDT value <80%, the combined effect was more likely to be less toxic than predicted by the dose-addition model (i.e., >90% frequency). In this study, at least one chemical of the combination always had a TDT value greater than 80%. There is a need for additional mixture testing in which both chemicals that have low TDT (i.e., <20%) to mid-range TDT values (i.e., 20–80%). Mixture toxicity analyses that incorporate differences in x/y curve slopes and chemical potencies, in addition to TDT data, offer the opportunity to improve prediction of chemical mixture toxicity.

## Conflict of interest

Nothing to declare.

## Transparency document

Transparency Document
